# Use of electrical impedance tomography to set positive end-expiratory pressure in a pediatric patient with severe acute respiratory distress syndrome

**DOI:** 10.31744/einstein_journal/2024CE0952

**Published:** 2024-09-25

**Authors:** Marcela Lopes Frade, Luciana Assis Pires Andrade Vale, Letícia Candançan Corrêa, Felipe de Souza Rossi, Celso de Moraes Terra, José Colleti

**Affiliations:** 1 Hospital Israelita Albert Einstein São Paulo SP Brazil Hospital Israelita Albert Einstein, São Paulo, SP, Brazil.; 2 Timpel Medical Company São Paulo SP Brazil Timpel Medical Company, São Paulo, SP, Brazil.

Dear Editor,

Pediatric acute respiratory distress syndrome is one of the main pediatric intensive care unit admission diagnoses.^(1)^ The adjustment of positive end-expiratory pressure (PEEP) to improve lung compliance and oxygenation is a key treatment strategy.^(2,3)^ However, there is a lack of evidence regarding the proper adjustment of PEEP during daily care.^(4,5)^

**Figure 1:**
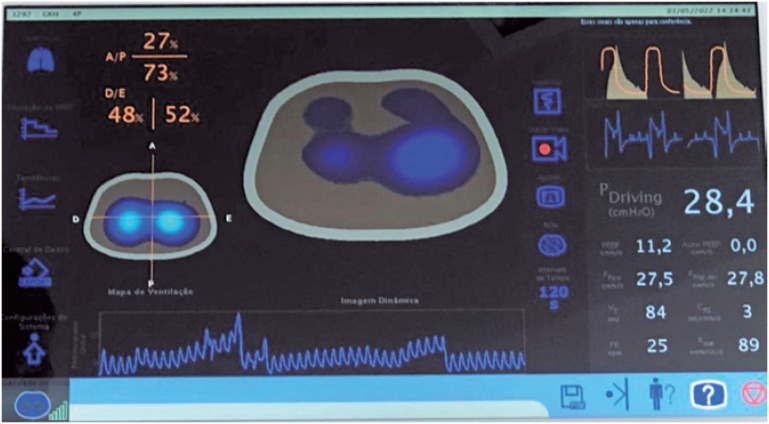
Electric impedance tomography to set PEEP

**Figure 2:**
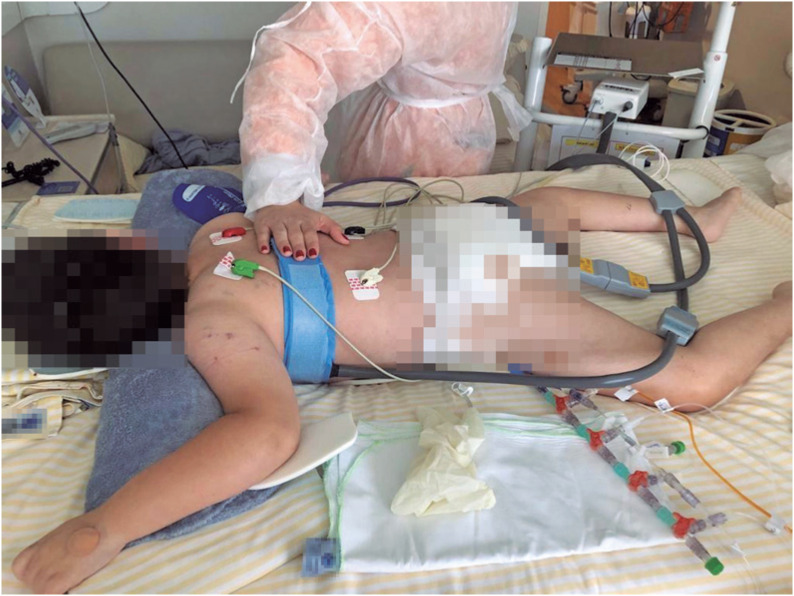
Patient in prone position with the electrical impedance tomography belt

To publicize a little-used strategy to set the ideal PEEP, we report a case of a 9-year-old patient admitted to our hospital with *Metapneumovirus* pneumonia who was mechanically ventilated due to severe acute respiratory distress syndrome. We used electrical impedance tomography in the prone position to set the PEEP for the patient (Figures 1 and 2), aiming to improve oxygenation for better lung compliance and to avoid hemodynamic compromise. Electrical impedance tomography revealed reduced oxygen requirements, which gradually improved. The patient was discharged on day 31 following admission.

It is important to alert clinicians that although there is a paucity of evidence on the best strategy to adjust PEEP in patients, the use of electrical impedance tomography seems promising.

The work was approved by the Research Ethics Committee of *Hospital Israelita Albert Einstein* (CAAE: 70860323.2.0000.0071; # 6.235.278).
